# Race, Ethnicity, and Social Engagement Among Community-Dwelling Older Adults: The Health and Retirement Study

**DOI:** 10.1093/geroni/igab046.098

**Published:** 2021-12-17

**Authors:** Lien Quach

**Affiliations:** University of Massachusetts Boston, Newton, Massachusetts, United States

## Abstract

Social engagement is crucial for older adults. This study examines the relationship between race, ethnicity, and social engagement among community-dwelling older adults using data came from the Health and Retirement Study (2014) (n=6221). Race and ethnic status were categorized as: non-Hispanic white (NHW), non-Hispanic black (NHB), non-Hispanic “Asians and other race” (NHA) and Hispanic (any race). Social engagement was based on self-report and included keeping in touch with friends, family and participating in social activities. Covariates included age, sex, education, number of comorbidities, physical function, and alcohol consumptions. The mean age was 74.6, 60% were female. Race and ethnicity distribution were 78.6% NHW, 11.9% NHB, 7.89% Hispanics, and 1.7% NHA. The social engagement (SE) score averaged 3.3. Hispanics, Asians and other races had a lower SE score compared with NHW (b=-0.29, p<.0001; b=-0.27, p=0.04). Understanding racial and ethnic disparities in SE can help target appropriate social intervention.

